# High normal TSH is associated with retinopathy and increased albumin excretion in euthyroid type 1 diabetic patients

**DOI:** 10.1186/1758-5996-7-S1-A22

**Published:** 2015-11-11

**Authors:** Leticia Maria Alcantara Margallo, João Batista Jornada Ben, Larissa Carolina Garcia Franco da Rosa, Marcus Miranda, Lenita Zajdenverg, Joana Dantas, Melanie Rodacki

**Affiliations:** 1 UFRJ, Rio de Janeiro, Brazil

## Background

Patients with Type 1 diabetes (T1D) have an increased risk of autoimmune diseases, especially thyroid disease, and untreated thyroid disease may interfere in the insulin sensitivity and glycemic control. Subclinical hypothyroidism and high levels of TSH have been associated with high risk of cardiovascular disease and chronic complications in type 2 diabetic (T2D) patients. TSH levels have also been linked to diabetic retinopathy and renal dysfunction in patients with T1D.

## Aim

The objective of this study was to evaluate the relationship between TSH levels and the prevalence of microvascular complications in euthyroid T1D patients with adequate glycemic control and at least 10 yrs. of disease.

## Materials and methods

This observational, retrospective study included patients with T1D for ≥ 10 yrs. without a known previous thyroid disease and a TSH measurement within the last year. Clinical and epidemiological data were obtained in an interview and the review of the medical charts. Patients were divided into two groups according to TSH levels: <2.5 mU/L and ≥2.5mU/l. Patients with TSH < 0.4 or ≥10 mU/L were excluded.

## Results

We included 118 individuals with a mean age of 27.84±9.25 yrs., mean disease duration of 17.11±7.13 yrs., mean HbA1c over the yrs. of 8.55±1.6% and mean current HbA1c of 8.32±1.64%. Thirty six patients (30.5%) had TSH≥2.5mU/l. There were no differences between groups stablished according to the TSH levels and HbA1c in the first 5 yrs. of disease (p=0.138), mean HbA1c over the years. (p=0.878) and current HbA1c (p=0.834). The prevalence of diabetic retinopathy was lower in those with TSH <2.5 mU/L than others (2.5% vs. 23.5%; p=0.024), as well as the prevalence of increased albumin excretion (7.3% vs. 50%; p<0.0001), however analyzing only patients with mean HbA1c over yrs. below 8.0%, this difference was not observed. The prevalence of diabetic neuropathy (peripheral and autonomic) were similar in both groups (p=0.41 and 0.103, respectively).

## Conclusion

TSH levels ≥2.5 mU/L are associated with a higher risk of diabetic retinopathy and increased albumin excretion in individuals with T1D with long duration of the disease. Further studies are necessary to identify if levothyroxine use might reduce the risk of complications in these individuals.

